# Stage of presentation and survival rates of head and neck cancer in Western Cape, South Africa

**DOI:** 10.4102/jcmsa.v2i1.2

**Published:** 2024-03-15

**Authors:** Simon M. Honnet, Oskar Edkins, Sameera Dalvie, Julie A. Wetter, Justin Harvey, Johannes J. Fagan

**Affiliations:** 1 Department of Surgery, Division of Otolaryngology, Faculty of Health Sciences, University of Cape Town, Cape Town, South Africa; 2 Department of Otolaryngology, Head and Neck Surgery, Townsville University Hospital, Townsville, Australia; 3 Department of Radiation Oncology, Division of Radiation Medicine, Faculty of Health Sciences, University of Cape Town, Cape Town, South Africa; 4 Department of Statistics and Actuarial Science, Faculty of Economics and Management Science, Stellenbosch University, Stellenbosch, South Africa

**Keywords:** head and neck, cancer, stage, overall survival, Western Cape, metropolitan, remote

## Abstract

**Background:**

The Western Cape province in South Africa has a high age-standardised mortality from cancer. Most literature reports significantly poorer access to healthcare, more advanced cancers and worse survival for rural and remote populations. This study investigates if significant disparities exist with regard to stage at presentation and overall survival of patients with head and neck cancer (HNC) between geographical areas within the Western Cape province in South Africa.

**Methods:**

A retrospective chart review was conducted on all patients managed at the Combined ENT & Head and Neck Oncology clinic at Groote Schuur from January 2010 to December 2014. Ethics approval was granted by the University of Cape Town Human Research Ethics Committee (HREC, 351-2017).

**Results:**

Although we observed no significant difference in TNM clinical stages or overall survival between metropolitan and remote patients, there were statistically and clinically significant differences in terms of both stages of HNC and overall survival between some individual metropolitan and remote areas. The remote area of Eden had a median overall survival of more than 6 months less than that of the Southern subdistrict of the City of Cape Town.

**Conclusion:**

Differences in HNC stages and overall survivals between some subdistricts of the City of Cape Town and remote areas are not only statistically significant but also clinically relevant.

**Contribution:**

This article highlights the need to improve on diagnostic and referral pathways for management of patients with HNC in the Western Cape.

## Introduction

Head and neck cancer (HNC) causes significant morbidity, by impacting bodily functions such as breathing, speech, swallowing and taste, as well as through the psychological and social impacts of the disease, and morbidity of treatment. Lower- and middle-income countries (LMICs) not only have the majority and increasing incidence of burden of cancer but also limited resources for its management.^1^

There is a wide geographical variation in the incidence of HNC, reflecting socioeconomic factors, and exposure to carcinogens such as smoking and alcohol. According to data from death certificates and from Statistics South Africa, the Western Cape province had substantially higher age-standardised mortality from cancers during our study period (118 per 100 000 population), which was close to double the national average of 69 per 100 000 population.^2^

Diagnosing HNC relies on a thorough history and specialised clinical examination. Management of HNC is therefore highly specialised and requires multidisciplinary input. With South Africa’s constrained financial and staffing resources, this requires that HNC services are centralised in metropolitan areas. The World Health Organization (WHO) Alma-Ata declaration in 1978 was the first, and now most well-known international declaration identifying the importance of primary healthcare (PHC) in attaining ambitious goals of ‘health for all’.^3^ Forty-four years on, many rural and remote areas of South Africa are severely underfunded and understaffed, and unable to provide adequate PHC.

Although some subsites of HNC do require specialised equipment for diagnosis, the majority do not. Tissue diagnosis is usually possible from a PHC clinic setting, for example, for oral cavity and oropharyngeal tumours because of the accessibility of the oral cavity and oropharynx for examination and biopsy. Laryngeal cancers are not as easily accessible for biopsy, but they do cause dysphonia even from an early stage. Laryngeal pathologies can be readily examined in a PHC clinic with a headlight and mirror, and flagged for urgent referral for specialist laryngoscopy and biopsy where necessary. In many cases, however, these early symptoms are neglected or overlooked by both patients and PHC workers.

Most literature reports significantly poorer access to healthcare, more advanced cancers, and worse survival in rural and remote populations. Coory used the Australian Standardised Geographic Classification, and Statistical Divisions to define capital cities, and found that rural and remote patients had higher mortality from prostate cancer.^4^ Jong found that patients with HNC from remote areas of Australia, as defined by the Accessibility/Remoteness Index had higher mortality.^5^ Olson reported that patients from rural populations, as defined by Canadian census data from the patients’ postal codes, presented with more advanced breast cancer, but had no difference in survival.^6^ Henley, using an arbitrary classification of population sizes of the counties in the United States (US), found age-adjusted disease-specific survivals for various cancers to be 14% worse in patients from rural areas.^7^ Onega estimated patients’ travel time by their US ZIP codes of the patients’ residences and geocoded point locations of their National Cancer Institute (NCI) Hospitals, as a proxy for rurality.^8^ They demonstrated significant burdens for access to specialised healthcare for non-urban populations.

It should, however, be observed that there are some contradictory data on this topic. Unger used the US’ Rural-Urban Continuum Codes that classify metropolitan areas according to their population, and non-metropolitan areas according to both population and proximity to metropolitan areas. They analysed a cohort of 36 995 patients with various cancers and found no significant difference in overall or disease-specific survival.^9^ Kim reported no significant difference in overall survival of HNC between rural and urban populations in Canada, using community size as a proxy for rurality.^10^

These apparent contradictions may be due in part to the semantic differences in ways that populations are defined; some researchers compared urban and rural populations, while others compared metropolitan and non-metropolitan populations. These comparisons are not necessarily equivalent. Definitions of what constitutes metropolitan, urban, suburban, and rural areas vary across different countries. ‘Remoteness’ in a country such as Australia may be very different from that in Western Europe. The European Commission for the World Bank has therefore called for harmonised definitions in this regard.^11^

Anecdotal evidence certainly suggests that remoteness from medical care and poor access to specialist medical centres are associated with advanced disease at presentation and poor overall survival.^12,13^

According to data from the 2011 Census,^14^ 36% of the Western Cape population live outside of the City of Cape Town metropolitan area, some in towns as large as George (population 114 000) and Paarl (population 112 000), and some in smaller towns and rural areas. Many of these non-metropolitan areas have good clinic and hospital infrastructure, but most of the surgery and oncology services for HNC are centralised in the City of Cape Town metropolitan area.

## Aims and objectives

To determine whether significant differences exist between metropolitan and remote geographical areas within the Western Cape province in South Africa, with regard to clinical stage at presentation or overall survival of patients with HNC managed with both curative and palliative intent.

## Research methods and design

A retrospective chart review was conducted of all patients presenting to the Combined ENT/Head and Neck Oncology Clinic at Groote Schuur Hospital in the 5-year period from January 2010 to December 2014 managed with both curative and palliative intent.

## Inclusion and exclusion criteria

Patients with squamous cell carcinomas of the oral cavity, oropharynx, larynx, hypopharynx, sinonasal cavities, salivary glands, skin and unknown primary sites were included in the study. Reasons for patient exclusions are outlined in Table 1.

**TABLE 1:** Reasons for patient exclusions.

Reason for exclusion	Number	%
Patients living outside of the Western Cape of South Africa	22	2.2
Patients with no South African identification documents	48	4.8
Incomplete records of the nature of their cancer site, type or staging	22	2.2
Patients with previous medical history of any cancer or radiation therapy	79	7.8
Patients developing cancer in non-head and neck areas during the follow-up period	5	0.5
Benign and premalignant tumours	34	3.3
Sarcoma, lymphoma, Merkel cell carcinoma, and neuroendocrine carcinoma	17	1.7
Nasopharyngeal carcinoma, carcinoma of the ear	42	4.1

Data from hospital folders were collected, collated, and reviewed by the first author. Demographic details such as patients’ age, gender, residential area and date of registration at the clinic were recorded, as well as cancer site, subsite, histology, and the TNM Classification of Malignant Tumors (TNM) staging.

**FIGURE 1:**
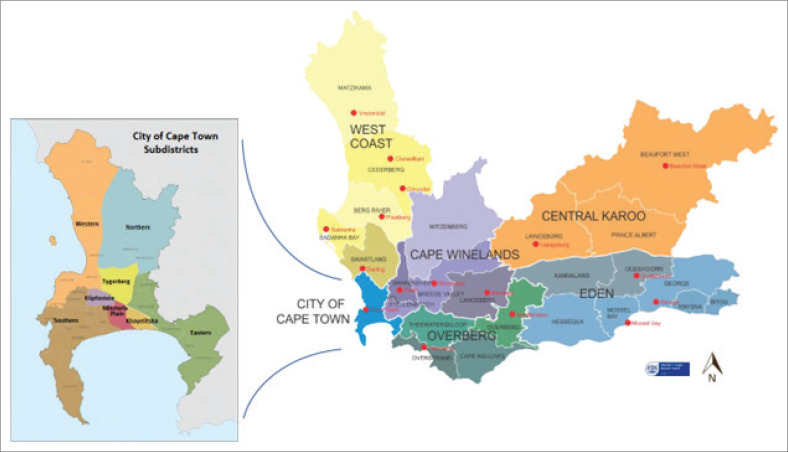
Residential areas, showing: City of Cape Town metropolitan subdistricts (boxed area) and remote districts of the Western Cape.

Residential areas, as defined by the Western Cape Municipality (Figure 1) included: City of Cape Town Metropole, with the following subdistricts: Northern, Southern, Eastern, Western, Tygerberg, Klipfontein, Mitchells Plain and Khayelitsha. The remainder of the Western Cape Province is divided into the following districts, which for the purpose of patient management, are remote: West Coast, Cape Winelands, Overberg, Eden, and Central Karoo.

Dates of death were in some cases recorded in the hospital records, but the majority were obtained from the open access registry at the Department of Home Affairs of South Africa. As the majority of patients had no record of the cause of death, we measured overall survival instead of disease-specific survival. We used a simple subtraction of the number of days between the date of registration with the clinic from the date of death to calculate the overall survival in days. The starting date was the first week of January 2010, when the first patients were registered and seen in the clinic, and data collection was terminated on 05 March 2020 because of the start of the coronavirus disease 2019 (COVID-19) pandemic in South Africa, as it had a major impact on survival of patients with comorbidities such as cancer.

All statistical analyses were performed in R (version 4.1.1). We used Kaplan–Meier curves to graphically demonstrate the cumulative survival, and log-rank tests for comparison of strata to determine independent predictors of overall survival, since crude survival figures do not take censored data into account. We also calculated median overall survivals and 5-year overall survivals for comparison to international literature.

### Ethical considerations

Ethics approval was granted by the University of Cape Town Human Research Ethics Committee, with approval number HREC, 351-2017. Anonymity of the patients’ records was preserved by allocation and use of a unique patient identification number in the spreadsheet of results, instead of their names, ID numbers, or hospital numbers.

## Results

### General epidemiology

A total of 1287 patient charts were reviewed and 269 were excluded for the reasons mentioned in Table 1. The remaining 1018 patients ranged in age from 8 to 103 years (median age 58.1 years) and included 714 males and 304 females (male-to-female ratio of 2.3:1).

Squamous cell carcinomas accounted for 94.3% of patients, and adenocarcinomas for 3.3%. Other subtypes of salivary carcinomas accounted for 1.1%, other sinonasal carcinomas for 0.8%, melanomas for 0.3%, and rare tumours for 0.2% of patients.

The oral cavity, larynx and oropharynx were the most common HNC sites, together accounting for 82% of cases. The remaining 18% were cancers of the sinonasal cavities, hypopharynx, major salivary glands, skin and HNC of unknown primary (HNCUP). There were only six patients with skin cancers, as the clinic only manages advanced cases requiring complex or major resections and reconstruction.

**FIGURE 2:**
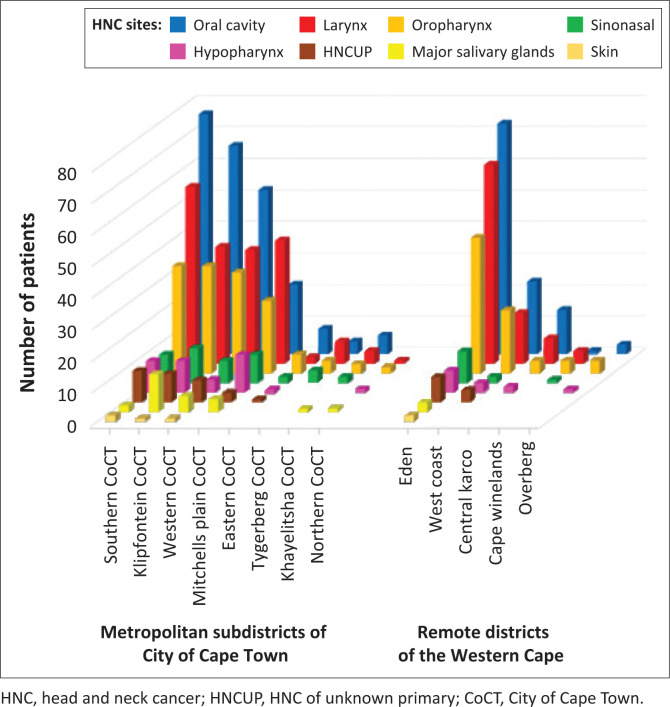
Burden of head and neck cancer in different referral areas.

Although all metropolitan and remote districts were represented, some areas like the Eastern, Northern, Tygerberg and Khayelitsha subdistricts of the City of Cape Town are served primarily by Tygerberg Hospital. Likewise, some areas of the Winelands and Malmesbury are served by Paarl and Tygerberg Hospitals, and some areas of the Overberg are served by Hottentots Holland and Tygerberg Hospitals (Figure 2).

All tumour sites had approximately double the number of patients from metropolitan areas compared to remote areas.

### TNM Stages according to geographical region

Most patients from both metropolitan (84%) and remote areas (88%) presented with advanced (stage III – IV) disease. There was no significant difference in Chi-squared analysis of the stages of cancer between all the metropolitan areas compared with all the remote areas (*p* = 0.16).

**FIGURE 3:**
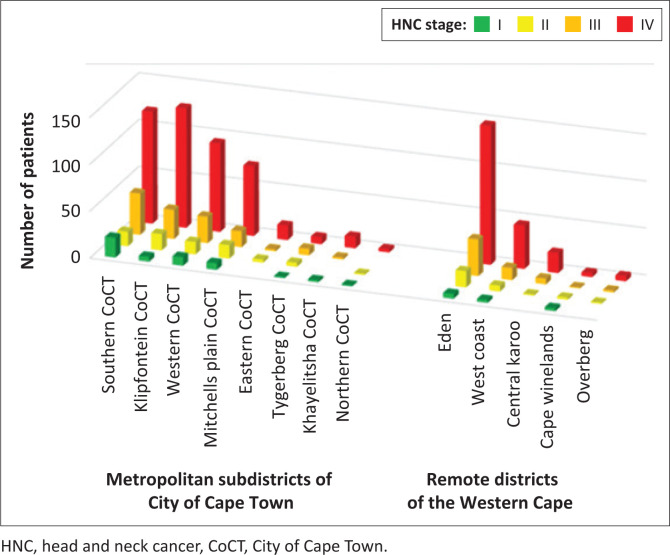
TNM Stage according to residential districts.

There were, however, some significant differences in cancer stages between different districts and subdistricts within these areas (*p* = 0.0123). Of stage I cancers, 37% originated in the Southern subdistrict of City of Cape Town, while all the remote areas *combined* had only 19% of stage I cancers. The Eden area (includes Oudtshoorn, George, Knysna, Plettenberg Bay and Mossel Bay) had a high burden of advanced (stages III and IV) cancers (187 cases); this represents 66% of all the remote areas’ advanced cancers (Figure 3).

### Overall survival according to geographical region

**FIGURE 4:**
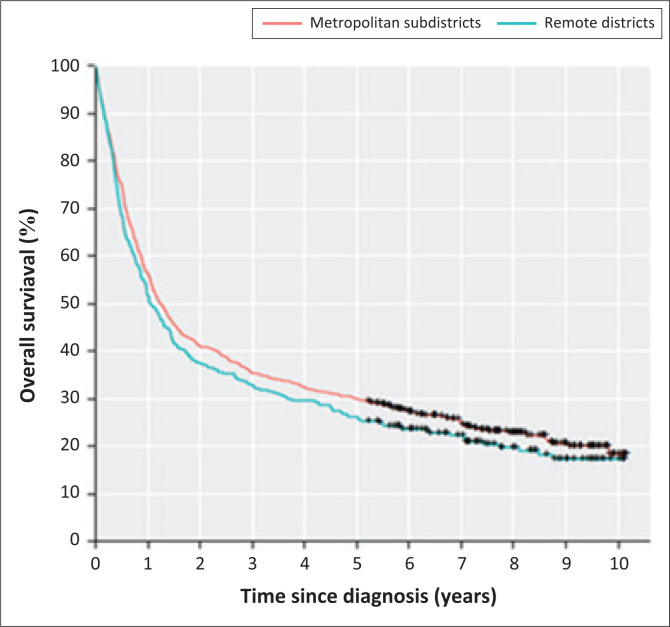
Overall survival according to metropolitan areas versus remote areas.

At the end of the study period, 231 of 1018 patients (22.7%) were still alive; for purposes of statistical analysis, these were recorded as censored data. The median follow-up time for these patients was 7.6 years. There was no significant difference in median overall survival between all the metropolitan areas combined, compared to all the remote areas combined (1.25 years vs. 1.06 years, *p* = 0.18) (Figure 4).

**FIGURE 5:**
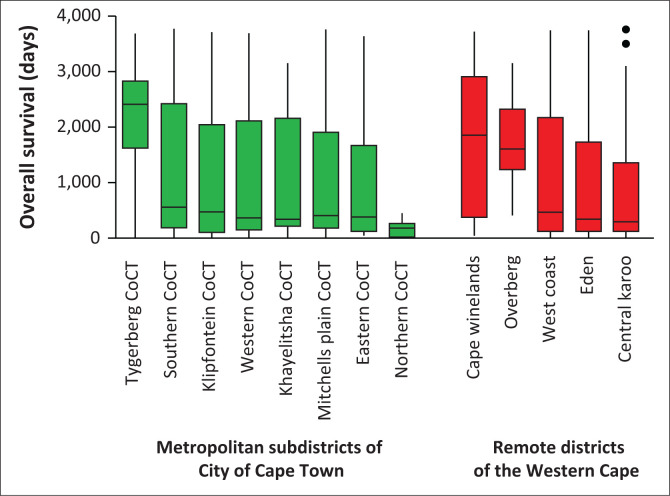
Median overall survival according to geographical region.

There were, however, significant differences in overall survival between specific metropolitan and remote districts (*p* = 0.015) (Figure 5). Although the City of Cape Town Tygerberg subdistrict, Cape Winelands and Overberg areas had much better overall survivals, and the City of Cape Town Northern Subdistrict had much worse overall survival, one should again bear in mind that these specific areas had a very low burden of disease being managed at the ENT/Head and Neck Oncology Clinic at Groote Schuur Hospital, possibly because of being served primarily by other hospitals.

We illustrated these clinically important differences in a box and whisker format, since the Kaplan–Meier graph had numerous overlapping lines making the interpretation difficult.

Atlantis is a town in a small part of the Western subdistrict of the City of Cape Town Metropolitan area, which had 52 patients with a similar median age (57.1 years), similar spectrum of HNCs, and the same proportion of advanced cancer (87%) as patients in the rest of the province. The median survival for patients from Atlantis was only 320 days, compared to 420 days for patients from the rest of the Western subdistrict of the City of Cape Town, and 477 days for patients from the rest of the subdistricts of City of Cape Town metropolitan areas. These apparent differences in survival were, however, not statistically significant (*p* = 0.133 and *p* = 0.079, respectively).

## Discussion

Advanced HNC is associated with significant morbidity related both to the disease and the treatment, and the long-term survival is poor, especially in LMICs like South Africa.^1,15^ Disparities in access to healthcare between high and LMICs are a well-known and long-standing public health concern, and are certainly not unique to South Africa. The Sustainable Development Goals aspire to achieving equitable access to healthcare; this includes not only geographical proximity but also economic affordability and cultural acceptability.^16^ This study describes the geographic distribution of the burden of HNC in the Western Cape, as well as demonstrating statistically and clinically significant differences in overall survival in specific metropolitan and remotes residential areas.

We measured overall survival of HNC patients managed with both curative and palliative intent, partly because of the limitations in our database and medical records, and partly since HNC recurrence usually occurs within the first few years after treatment,^17^ as such the two measures of survival should be similar in the context of our follow-up period. Intuitively, one may assume that disease-specific survival would be more relevant to assess cancer mortality, but this is not necessarily so. Non-surgical treatment of advanced HNC is often complicated by a high risk of micro-aspiration and recurrent chest infections. In LMICs without social service support, patients may become impoverished by having to pay for surgery^18^ or because of the sequelae of the cancer, or of its treatment. Therefore, if the underlying cause(s) of death are not interrogated, disease-specific survival may under-report the true mortality of the disease *and* its treatment. Overall survival, on the other hand, considers death by any cause.

The clinical stage of HNC at presentation is an important prognostic factor for HNC, independent of factors such as age, gender, and tumour location.^19,20^ Early diagnosis of HNC allows shorter durations of treatment,^21^ lower morbidity^22^ and better prognosis^23,24,25,26^; advanced stages of HNC limit treatment options, and reduce overall survival.^27^ Organ-preservation treatment of HNC is optimal if the diagnosis is made early. The results in this study certainly support this literature. The Southern subdistrict of City of Cape Town (with the most stage I HNCs) had a median overall survival of 550 days, while the remote Eden area (with two thirds of the remote areas’ advanced HNCs) had a median overall survival of only 357 days. Atlantis in the Western subdistrict had a median survival of only 320 days, which may partly reflect its remoteness, since public transport between Atlantis and Woodstock was only introduced in April 2014, towards the end of our study period. It is also likely that this reflects the extremely poor socioeconomic circumstances in the area, with high levels of unemployment, poverty, homelessness, and malnutrition.

Delays in treatment, especially for some sites of HNC such as cancers of the oral cavity, oropharynx, and larynx, are therefore particularly tragic because most of the oral cavity and oropharynx can be viewed and palpated without special instrumentation, and laryngeal cancers generally present with voice symptoms at an early stage.

Our data show that a substantial portion (17%) of stage IV HNCs had an overall survival of greater than five years, showing that even advanced HNCs do not necessarily have uniformly poor outcomes.

Even though we found no significant difference in median overall survival between all the metropolitan areas combined, compared to all the remote areas combined (*p* = 0.18), there were significant differences in overall survival between specific metropolitan and remote districts (*p* = 0.015).

One of the difficulties hampering comparative research in this field is the lack of standardised definitions as to exactly what constitutes ‘urban’ and ‘rural’, or ‘metropolitan’ and ‘non-metropolitan’ populations. Wikipedia defines ‘urban’ as being a built-up area with high population density and infrastructure of built environment, and ‘rural’ as having low population density, often with agricultural or forestry land usage. On the other hand, a metropolis is any major city, together with its nearby towns and environs. In South Africa (and many other countries), it would be an over-simplification for this type of research to categorise populations simply as urban and rural alone because there is a broad continuum between these two extremes. For example, many South African shanty towns have sufficient population densities to be classified as urban but lack any economic core to be functionally urban. Likewise, many formally structured small towns are so geographically remote, that they cannot be truly urban.

One can also use the distance between towns and hospitals to assess remoteness, but again, for a small town 100 km from the hospital, this may be quite remote for a patient using a week’s wages in travel costs but may not be so remote for a patient who uses their own transport.

It is difficult to determine what factors negatively affect outcomes in remote, rural or non-metropolitan populations. It is likely not only the longer distances that deter travel to tertiary hospitals but also lower levels of formal education, lower socioeconomic status, greater poverty and malnutrition, higher levels of smoking and alcoholism, different attitudes towards healthcare, and cultural practices, for example, traditional healthcare, which also play a role.^28,29,30,31^ It is also likely that these same factors are associated with more undiagnosed and untreated comorbidities, reducing general life expectancy, which impacts to some extent on overall survival.

## Strengths and limitations of the study

The strengths of this study are the large sample size of 1018 consecutive patients, all of whom were managed by the same multidisciplinary medical team, and the long duration of their follow-up of 7.6 years (of patients still alive at the end at the end of our study period). The weaknesses are the absence of measures of socioeconomic status, which may be more relevant than a patient’s address. Future studies should investigate this, as well as prospectively enquire as to the underlying cause of delays in diagnosis at PHC levels, and to determine the cause of death. Future studies may also identify whether treatment intents are curative or palliative, and analyse these subgroups separately.

## Conclusion

The bulk of HNC managed at the Groote Schuur HNC clinic originates from the City of Cape Town Metropole; yet there is a significant burden of disease in the remote Eden area (includes Oudtshoorn, George, Knysna, Plettenberg Bay and Mossel Bay), and in Atlantis in the Western subdistrict of the Cape Town metropolitan area. The difference in median overall survivals between the Southern subdistrict of City of Cape Town (550 days) and Eden (357 days) is not only statistically significant but is also clinically relevant. This highlights the need to improve on diagnostic and referral pathways. Despite these disparities at a district level, there was no significant difference in TNM clinical stages or overall survival between metropolitan and remote patients.

While there are significant differences between some metropolitan and remote areas in terms of clinical stage at presentation and overall survival, this is likely because of many factors, such as socioeconomic factors and other hospitals serving some of these areas.
